# A biparatopic agonistic antibody that mimics fibroblast growth factor 21 ligand activity

**DOI:** 10.1074/jbc.RA118.001752

**Published:** 2018-02-26

**Authors:** Sally Yu Shi, Ya-Wen Lu, Zhi Liu, Jennitte Stevens, Christopher M. Murawsky, Vicki Wilson, Zhonghua Hu, William G. Richards, Mark Leo Michaels, Jun Zhang, Wei Yan, Yang Li

**Affiliations:** From ‡ Amgen Inc., South San Francisco, California 94080,; § Amgen Inc., Thousand Oaks, California 91320, and; ¶ Amgen British Columbia Inc., Burnaby, British Columbia V5A 1V7, Canada

**Keywords:** drug development, antibody engineering, single-domain antibody (sdAb, nanobody), fibroblast growth factor (FGF), fibroblast growth factor receptor (FGFR), biparatopic antibody, bispecific antibody, β-Klotho

## Abstract

Bispecific antibodies have become important formats for therapeutic discovery. They allow for potential synergy by simultaneously engaging two separate targets and enable new functions that are not possible to achieve by using a combination of two monospecific antibodies. Antagonistic antibodies dominate drug discovery today, but only a limited number of agonistic antibodies (*i.e.* those that activate receptor signaling) have been described. For receptors formed by two components, engaging both of these components simultaneously may be required for agonistic signaling. As such, bispecific antibodies may be particularly useful in activating multicomponent receptor complexes. Here, we describe a biparatopic (*i.e.* targeting two different epitopes on the same target) format that can activate the endocrine fibroblast growth factor (FGF) 21 receptor (FGFR) complex containing β-Klotho and FGFR1c. This format was constructed by grafting two different antigen-specific VH domains onto the VH and VL positions of an IgG, yielding a tetravalent binder with two potential geometries, a close and a distant, between the two paratopes. Our results revealed that the biparatopic molecule provides activities that are not observed with each paratope alone. Our approach could help address the challenges with heterogeneity inherent in other bispecific formats and could provide the means to adjust intramolecular distances of the antibody domains to drive optimal activity in a bispecific format. In conclusion, this format is versatile, is easy to construct and produce, and opens a new avenue for agonistic antibody discovery and development.

## Introduction

Monoclonal antibodies are an important drug class for treating human disease. With advantages of prolonged pharmacokinetic properties, target specificity, well-established discovery technologies, and continued improvements in cost-effective manufacturing, they have become the preferred class of molecules for biologic therapeutic development. While historically antibody drug development programs have focused on antibodies that bind a target through engaging a single epitope site, bispecific (or even multispecific) antibodies have gained significant attention in recent years. Bispecific molecules allow for synergy by engaging two separate targets, or result in superior properties by engaging two epitopes on the same target (1, 2). In addition, bispecific antibodies may enable new functions that are not possible to achieve with monospecific antibodies alone, such as tissue-specific delivery or bringing cytotoxic T cells to tumor sites (1, 2).

The strong interest in bispecific molecules has fueled tremendous innovation and over 100 different formats have now been described, including bispecific IgG, numerous fusions to IgG, and the use of various antibody fragments (1, 2). This array of different formats provides flexibility for a fit-for-purpose design based on size, valency, geometry of antigen-binding sites, pharmacokinetics, and additional properties such as effector functions. However, compared with traditional IgGs, which consist of one heavy and one light chain, bispecific antibodies are more complex, and may result in difficulties in expression and complications in manufacturing that could affect the cost of the drug product. Although many of the novel antibody formats were designed to address some of the challenges associated with bispecific antibodies, there is room for further improvements.

Since the introduction of monoclonal antibody drugs more than three decades ago, tremendous technological advances have been made to the drug discovery and development process. The properties of monoclonal antibodies are well understood, and the chemistry, manufacturing, and control process is well established and cost-effective. Therefore, our bispecific antibody design rationale was to take advantage of these robust platforms and engineer a bispecific antibody that employs a scaffold similar to that of a traditional monoclonal antibody.

Several approaches have been described to generate a bispecific molecule that resembles a traditional monoclonal antibody in architecture. For example, the “knobs-into-holes” modification in the CH3 domain allows for the heterodimerization between two antibody arms that bind two different antigens (3). A major hurdle with this approach is the potential mispairing of light chains with inappropriate heavy chains leading to reduced yield of the correct product and, subsequently, a more challenging manufacturing process (1). Alternative approaches, such as common light chains, have been explored to circumvent the mispairing issue (4). To further eliminate the need for two different heavy chains in an IgG setting, the engineering of a single variable domain heavy chain (VH)^3^ and variable domain light chain (VL) pair to bind two different antigens has been reported (5). This bispecific format is reminiscent of a traditional IgG and is constructed by using the antibody against the first antigen as the base to generate a library of light chain pairings that are then screened for their ability to bind a second antigen.

Here, we explored a slightly different approach that takes advantage of the unique properties of single-domain antibodies in therapeutic drug development. First discovered in camelids, VHHs are single-domain antibodies made of the variable region of a heavy chain without a cognate light chain (6). In addition to their small size, single-domain antibody fragments offer numerous advantages such as ease of genetic manipulation and production, amenability to the engineering of multivalent formats, increased functional size of immune libraries, and high solubility and physicochemical stability. We used a transgenic antibody discovery platform, the Harbour heavy chain-only antibody (HCAb) mouse, to generate fully human, antigen-specific VH domains that are functionally analogous to VHH domains (7). Our bispecific format grafts two different human VH domains that bind two distinct targets onto the CH1 and CL domains of the heavy and light chains, respectively, generating a bispecific molecule that requires neither knobs-into-holes nor sequential screening of a compatible light-chain binder to yield bispecificity.

We thus took advantage of the flexibility of the VH domains to explore various formats and geometries to optimize agonism of the β-Klotho/FGFR1c receptor complex to mimic the actions of fibroblast growth factor (FGF) 21. FGF21 belongs to the unique FGF19 subfamily of FGFs that functions as an endocrine hormone and plays important roles in metabolic regulation (8, 9). FGF21 lowers serum glucose, triglyceride, and cholesterol levels; improves insulin sensitivity; and reduces body weight in many diabetic rodent and nonhuman primate models (10). The associated benefits have prompted considerable interest in developing therapeutics against this pathway (11, 12). FGF21 signals through FGF receptors (FGFRs) and the interaction between FGF21 and FGFR occurs only in the presence of the co-receptor, β-Klotho, a single-pass transmembrane protein with two homologous extracellular domains (13, 14). Two different kinds of agonistic antibodies have been described to activate the β-Klotho/FGFR1c receptor complex and therefore mimic FGF21 actions (15–17). One example of an agonistic antibody is a traditional monoclonal antibody identified through immunization campaigns in Xenomouse® (15, 17). The other is a bispecific antibody consisting of one arm binding to β-Klotho and the other to FGFR1c, assembled together using the knobs-into-holes approach (16). Although these antibodies demonstrated the technical feasibility of activating β-Klotho/FGFR1c with antibody molecules, given the large distance between the two arms of both formats compared with the native ligand, it is likely that the receptor activation mechanism is different from the endogenous ligand. For antagonistic antibodies, which represent the vast majority of the bispecific molecules in development (1), the relative geometry of the binding modules is less critical. Given that agonists need to faithfully mimic the activity of the natural ligand, the binding geometry could be crucial (17, 18). Because our biparatopic molecule has a different geometry compared with hetero-Ig–based bispecifics (16), we sought to test its ability to activate the β-Klotho/FGFR1c signaling pathway. Herein, we demonstrate that single-domain antibodies can be put together to drive agonistic activity. Further, we demonstrate that our tetravalent, biparatopic format binds two distinct epitopes on β-Klotho, eliminates product heterogeneity due to multiple chains, and can activate the β-Klotho/FGFR1c complex with superior activity than each paratope alone. Thus, this approach offers the potential to address the heterogeneity concerns with other bispecific formats as well as a novel way to adjust intramolecular distances of the antibody domains to drive optimal activity in a bispecific format.

## Results

### Identification of heavy chain–only domains that interact with β-Klotho

**Figure 1.**
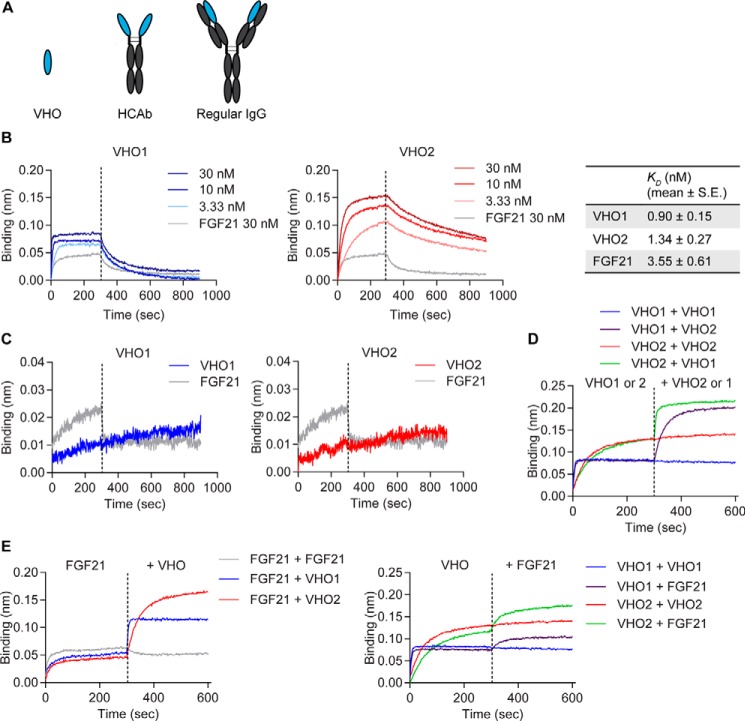
**Identification of heavy chain-only domains that interact with human β-Klotho.** *A*, schematic representation of the structures of VHO, HCAb, and a traditional IgG antibody. *B*, binding of VHO1 and 2 to human β-Klotho measured by bio-layer interferometry. *K_D_* values were estimated using a globally fitted 1:1 binding model. Data are mean ± S.E. (*n* = 3). *C*, binding of VHO1 and 2 (30 nm) to human FGFR1c measured by bio-layer interferometry. *D*, competition between VHO2 and VHO1 for β-Klotho binding. Human β-Klotho ECD His_6_ protein was immobilized onto biosensors in an Octet RED instrument and exposed to VHO1 and VHO2 (30 nm) in two consecutive association steps. *E*, competition between VHOs and FGF21 for β-Klotho binding. Human β-Klotho ECD His_6_ protein was immobilized onto biosensors in an Octet RED instrument and exposed to FGF21 (30 nm) and VHO1 or 2 (30 nm) in two consecutive association steps. Data are representative of three independent experiments.

VH domains recognizing human β-Klotho were generated from the Harbour mouse as described in “Experimental procedures.” Two of the recovered VHs (VH1 and VH2) were then cloned into expression vectors in two different formats, as VH only (VHO) or as HCAb (Fig. 1*A*). The affinities of the two VHO binders toward β-Klotho were measured using bio-layer interferometry on an Octet system. The dissociation constants (*K_D_*) determined for VHO1 and VHO2 were 0.90 nm and 1.34 nm, respectively, whereas the *K_D_* for FGF21 was 3.55 nm (Fig. 1*B*). In addition, the interaction of the two VHOs with the extracellular domain (ECD) of FGFR1c was also measured. In contrast to results obtained with β-Klotho, no detectable interaction was observed between either VHO and FGFR1c (Fig. 1*C*). FGF21 showed weak binding to the FGFR1c ECD in the absence of β-Klotho (Fig. 1*C*), consistent with previous observations (15). To better understand if the two VHOs bind to distinct regions on β-Klotho, we performed cross-competition studies. β-Klotho ECD was immobilized onto a biosensor and presented to the two VHOs in consecutive association steps (Fig. 1*D*). When the same VHO was used in both association steps, the second binding phase showed a minimal increase in signal, suggesting a near-complete saturation of binding sites on β-Klotho. In contrast, binding of VHO1 in the first association step failed to block subsequent binding of VHO2. The same result was obtained when the assay was run in reverse (VHO2 first, VHO1 second), indicating that VHO1 and VHO2 bind distinct and nonoverlapping epitopes on β-Klotho. Importantly, using a similar assay, we did not observe competition between either β-Klotho–binding VHO molecule and FGF21 for binding to β-Klotho (Fig. 1*E*). Neither VHO1, VHO2, or FGF21 associated nonspecifically with the streptavidin sensors, and the VHOs did not interact directly with each other or with FGF21 (data not shown). Together, these results indicate that VHO1, VHO2, and FGF21 bind to distinct regions on β-Klotho.

### Bivalency of heavy chain–only domains is required to activate receptor signaling

**Figure 2.**
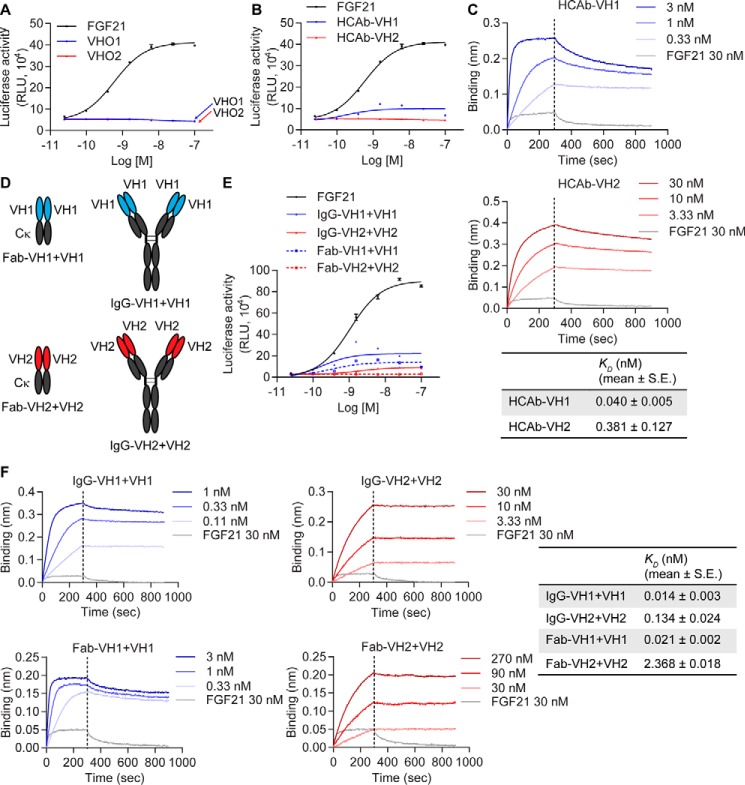
**Bivalency of heavy chain-only domains is required to activate receptor signaling.** *A* and *B*, stimulation of Elk1-luciferase activity by FGF21 (*A*), VHO1 and VHO2 and (*B*) HCAb-VH1 and HCAb-VH2 in CHO reporter cells stably expressing human β-Klotho and FGFR1c. *C*, binding of HCAb-VH1 and 2 to human β-Klotho measured by bio-layer interferometry. *K_D_* values were estimated using a globally fitted 1:1 binding model. *D*, schematic representation of IgG-VH1+VH1, IgG-VH2+VH2, Fab-VH1+VH1, and Fab-VH2+VH2. *E*, stimulation of Elk1-luciferase activity in CHO reporter cells. *F*, binding of antibody molecules to human β-Klotho measured by bio-layer interferometry. *K_D_* values were estimated using a globally fitted 1:1 binding model. All data are representative of three independent experiments and expressed as mean ± S.E.

The ability of the β-Klotho–binding VH domains to modulate receptor activity was determined using an FGF21-responsive reporter cell assay in CHO cells that stably express both human β-Klotho and FGFR1c. The co-expression of reporter constructs encoding a 5× Upstream Activation Sequence upstream of luciferase and Gal4 DNA-binding domain fused to Elk1 allows us to follow the ability of FGF21 or other test molecules to stimulate β-Klotho–dependent FGFR downstream signaling as a measure of luciferase activity (19). Although the VHs in the VHO format did not activate receptor signaling (Fig. 2*A*), VH1 in the HCAb format (HCAb-VH1) showed weak partial agonistic activity (Fig. 2*B*) (18.9 ± 5.2% *versus* 6.8 ± 2.6% of FGF21 *R*_max_ for HCAb-VH1 and HCAb-VH2, respectively, *p* = 0.02). This result demonstrates that bivalency is required for VH1 to activate the receptor complex, consistent with the general belief that dimerization of FGF receptors is required for activation of receptor tyrosine kinase activity (20). Bivalency also improved the functional binding affinity of VH1 for β-Klotho, as evidenced by a 20-fold reduction in *K_D_* over the VHO format (Fig. 2*C*). However, HCAb-VH2 remained inactive in the reporter assay (Fig. 2*B*), despite displaying an improvement in binding avidity over the monovalent VHO2 format (Fig. 2*C*).

To understand the impact of geometry on the agonistic activities of the two VHs, two other multivalent formats were also explored. As depicted in Fig. 2*D*, instead of fusing the VHs onto the Fc fragment as in the case of the HCAb format, we grafted the individual VH domains onto the CH1 and CL domains of the heavy and light chains, respectively. This can be done to produce a bivalent Fab (Fab-VH1+VH1 or Fab-VH2+VH2) (Fig. 2*D*) or a tetravalent monoparatopic IgG (IgG-VH1+VH1 or IgG-VH2+VH2) (Fig. 2*D*). These formats result in molecules having both close (the two arms in a Fab) and more distant (the two binding arms in the IgG) geometries between the different binding arms. As shown in Fig. 2*E*, whereas Fab-VH1+VH1 activated receptor signaling to a level similar to that observed with the HCAb-VH1 molecule, the tetravalent IgG-VH1+VH1 exhibited higher activity (25.7 ± 1.2% *versus* 18.6 ± 1.6% of FGF21 *R*_max_ for IgG-VH1+VH1 and Fab-VH1+VH1, respectively, *p* = 0.02). This suggests that the relative geometry and potential synergy between the two binding arms in the IgG format resulted in a more favorable receptor conformation for signaling. Consistent with this, although Fab-VH2+VH2 remained inactive similar to HCAb-VH2, the tetravalent IgG-VH2+VH2 modestly activated receptor signaling (Fig. 2*E*) (10.4 ± 0.3% of FGF21 *R*_max_). Of note, IgG-VH1+VH1 exhibited an inverted U-shaped dose-response curve, suggesting inhibition of signaling activity at high concentrations. This likely resulted from the multiple VH1 domains competing with each other for β-Klotho–binding sites when the concentration was high. Compared with HCAbs, the tetravalent IgG format was associated with a further increase in binding affinity and avidity for β-Klotho (Fig. 2*F*).

### Generation of a bispecific biparatopic antibody

**Figure 3.**
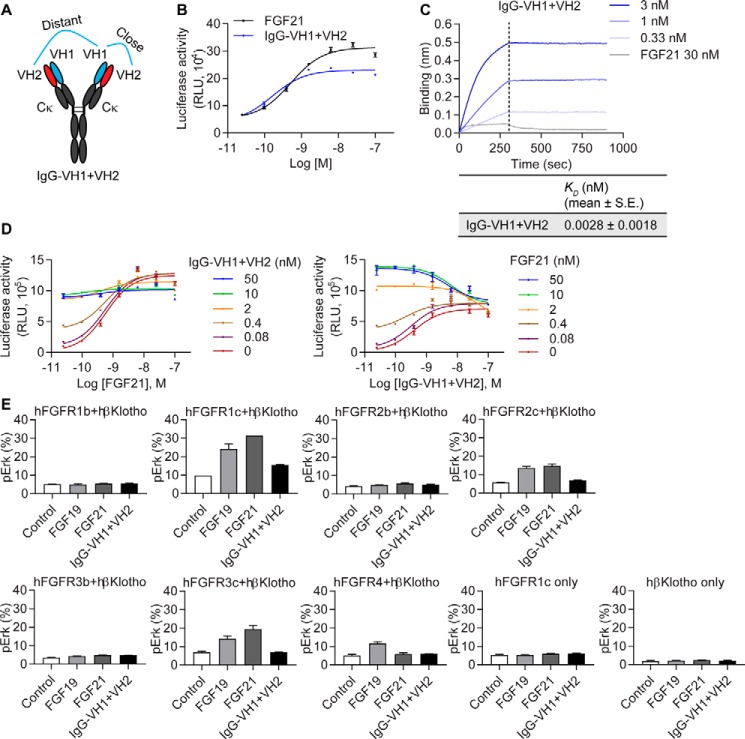
**Generation of a biparatopic monoclonal antibody.** *A*, schematic representation of the biparatopic antibody, IgG-VH1+VH2. The close and distant geometries of the two β-Klotho binding domains are noted. *B*, stimulation of Elk1-luciferase activity in CHO reporter cells. *C*, binding of IgG-VH1+VH2 to human β-Klotho measured by bio-layer interferometry. *K_D_* values were estimated using a globally fitted 1:1 binding model. *D*, inhibition of FGF21 signaling by IgG-VH1+VH2. CHO reporter cells stably expressing human β-Klotho and FGFR1c were treated with a combination of FGF21 and IgG-VH1+VH2 for 4 h, after which luciferase activity was measured. *E*, rat L6 cells were co-transfected with expression vectors encoding human FGFRs and β-Klotho. Cells were stimulated with treatment molecules (100 nm) followed by analysis of cell lysates for pERK. Results are expressed as percentage of phosphorylated ERK protein over total ERK protein. Data are representative of three independent experiments and expressed as mean ± S.E.

Although dual epitope targeting of a single protein has been shown to provide superior properties in the case of antagonists (1), this has not been explored for agonists. Because VH1 and VH2 bind different epitopes on β-Klotho, we were interested in the effect of combining them on receptor signaling activation. In addition, the design of the biparatopic antibody format allows us to address the role of binding domain geometry with regard to agonism (Fig. 3*A*). Our biparatopic IgG format generally resembles that of a typical antibody and is an extension of the monoparatopic IgG format shown in Fig. 2*D*, where the heavy chain consists of a VH1 fusion to the CH1-CH3 domains and the light chain consists of a VH2 fusion to the CL domain of the Cκ light chain. The expression and purification of this biparatopic antibody (named IgG-VH1+VH2) was carried out in a manner similar to a conventional antibody without the concern of potential mispairing between the heavy and light chains as has been observed with other bispecific formats (1).

In FGF21-responsive CHO reporter cells expressing human β-Klotho and FGFR1c, IgG-VH1+VH2 activated receptor signaling with potency similar to that of FGF21 (EC_50_ = 0.5 nm) (Fig. 3*B*). The maximal response achieved was ∼75% of that of FGF21, demonstrating superior efficacy than VH1 and VH2 in either the HCAb, monoparatopic Fab, or IgG format (compare Fig. 3*B* to Fig. 2, *B* and *E*). Characterization of the interaction between IgG-VH1+VH2 and β-Klotho by bio-layer interferometry indicated that IgG-VH1+VH2 directly interacted with β-Klotho with a much higher affinity than that of FGF21 (*K_D_* of 2.8 pm *versus* 3.5 nm, respectively) (Fig. 3*C*).

As neither arm of the biparatopic IgG molecule shares the same epitope with FGF21 on β-Klotho (Fig. 1*E*), we asked whether IgG-VH1+VH2 and FGF21 can act cooperatively to activate β-Klotho/FGFR1c signaling. To this end, we assessed the effect of different concentrations of IgG-VH1+VH2 on the dose response of FGF21, and vice versa, using FGF21-responsive reporter cells. As shown in Fig. 3*D*, IgG-VH1+VH2 inhibited FGF21 signal activation in a dose-dependent manner, with the maximal inhibitory response converging with the intrinsic activity observed with IgG-VH1+VH2 alone. These results indicate that IgG-VH1+VH2 antagonized the activity of FGF21. Because VHO1 or 2 alone did not compete with FGF21 for β-Klotho binding (Fig. 1*E*), we postulated that the receptor complex may be cross-linked together by the tetravalent biparatopic antibody, which makes the FGF21-binding sites on β-Klotho inaccessible, thereby inhibiting FGF21-induced signaling.

We next assessed whether IgG-VH1+VH2, being a strong binder to β-Klotho, can activate other FGFRs. To this end, we utilized the rat myoblast L6 cell line, which typically does not respond to FGF treatment because of low or no expression of endogenous FGFRs (15). As such, this system allowed us to study the downstream signaling of specifically reconstituted FGF receptors. L6 cells were co-transfected with human β-Klotho and each of the human FGFR1b, FGFR1c, FGFR2b, FGFR2c, FGFR3b, FGFR3c, and FGFR4 receptors, and pERK levels were measured after treatment with FGF21, FGF19, or IgG-VH1+VH2. Consistent with previous reports (15), FGF19 and 21 activated only the c isoforms of FGFR1, 2, and 3 in complex with β-Klotho, whereas FGF19, but not FGF21, activated the FGFR4/β-Klotho complex (Fig. 3*E*). In contrast, although the two arms of IgG-VH1+VH2, VH1 and VH2, do not interact with FGFR1c directly (Fig. 1*C*), it demonstrated specificity toward the β-Klotho/FGFR1c complex and did not activate the other FGFR receptors (Fig. 3*E*). In addition, the activity of IgG-VH1+VH2 was dependent on the presence of both FGFR1c and β-Klotho, as transfection with either receptor alone did not lead to ERK phosphorylation (Fig. 3*E*).

**Figure 4.**
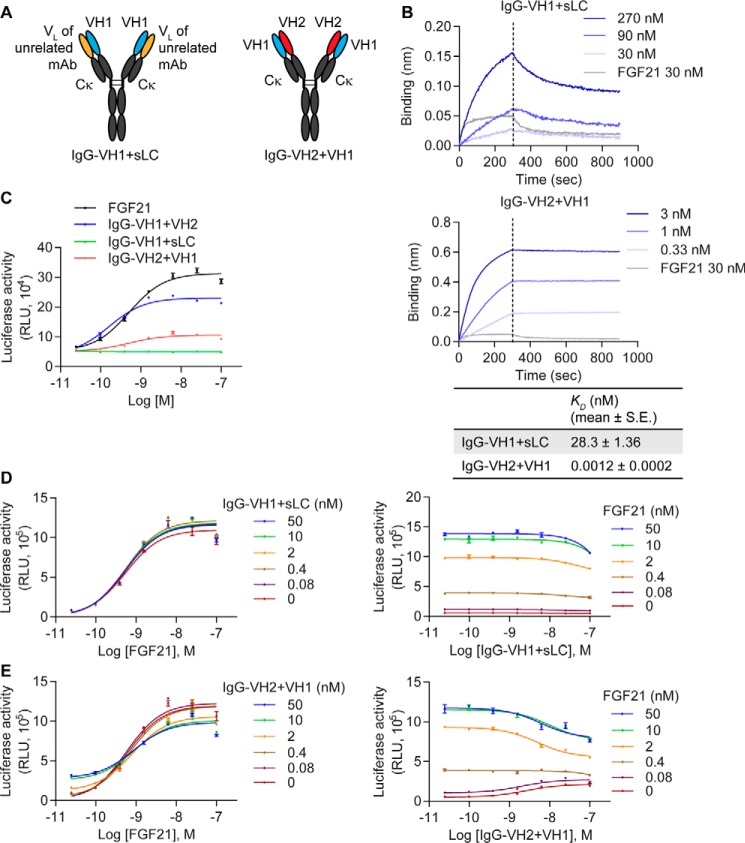
**The correct orientation of VH1 and VH2 domains is required for optimal agonistic activity.** *A*, schematic representation of IgG-VH1+sLC (sLC represents a light chain from an unrelated antibody) and IgG-VH2+VH1. *B*, binding of IgG-VH1+sLC and IgG-VH2+VH1 to human β-Klotho measured by bio-layer interferometry. *K_D_* values were estimated using a globally fitted 1:1 binding model. *C*, stimulation of Elk1-luciferase activity in CHO reporter cells. *D* and *E*, effect of (*D*) IgG-VH1+sLC and (*E*) IgG-VH2+VH1 on FGF21 signaling. CHO reporter cells stably expressing human β-Klotho and FGFR1c were treated with a combination of FGF21 and antibody molecules for 4 h, after which luciferase activity was measured. Data are representative of three independent experiments and expressed as mean ± S.E.

Because neither VHO2 nor HCAb-VH2 activated receptor signaling, we wondered whether the VH2 portion contributed to the agonistic activity of IgG-VH1+VH2. To test this, we replaced the VH2 portion of IgG-VH1+VH2 with the VL domain of a kappa light chain from an unrelated antibody (Fig. 4*A*). This new IgG molecule (named IgG-VH1+sLC) showed attenuated binding to β-Klotho compared with IgG-VH1+VH2 (Fig. 4*B*). It also did not activate FGFR1c signaling in the CHO reporter cells (Fig. 4*C*) nor inhibit FGF21 activity (Fig. 4*D*), demonstrating that the full agonistic activity of the biparatopic antibody, IgG-VH1+VH2, required the presence of both VH1 and VH2 arms.

To shed light on whether the relative orientation of the two VH arms is important for β-Klotho/FGFR binding and activation, we swapped the positions of VH1 and VH2. Namely, we placed VH2 on the heavy chain and VH1 on the light chain (IgG-VH2+VH1) (Fig. 4*A*). Similar to the original biparatopic molecule, IgG-VH2+VH1 could bind to β-Klotho with picomolar affinity (Fig. 4*B*). However, the agonistic activity of this molecule was significantly attenuated, achieving only one third of the maximal response of IgG-VH1+VH2 (Fig. 4*C*) (75.7 ± 2.2% *versus* 29.0 ± 4.2% of FGF21 *R*_max_ for IgG-VH1+VH2 and IgG-VH2+VH1, respectively, *p* = 0.0001). This result suggests that the geometry of the two VH domains is crucial for optimal receptor activation. Different orientations of VH1 and VH2 may affect the ability of β-Klotho to interact with FGFR1c, which in turn influences FGFR1c dimerization and downstream signal transduction. In line with this, and in contrast to the original biparatopic molecule, IgG-VH2+VH1 only partially inhibited FGF21 signaling in CHO reporter cells (Fig. 4*E*).

**Figure 5.**
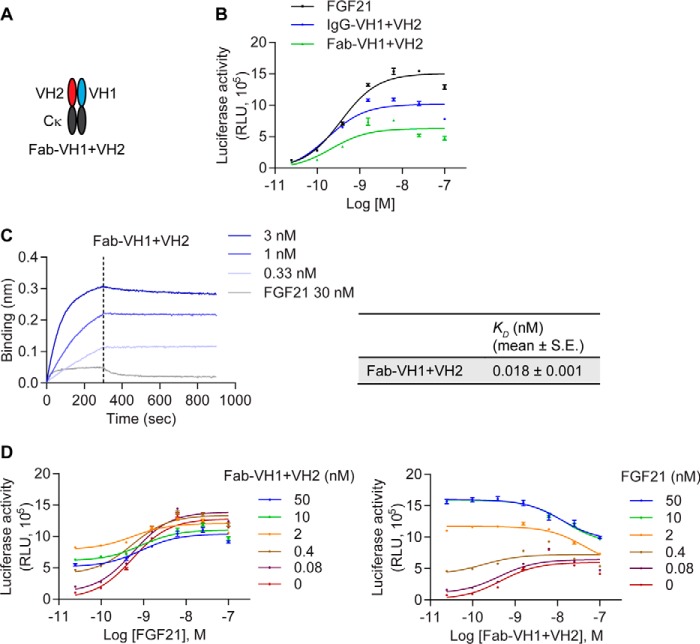
**The Fab fragment of IgG-VH1+VH2 can activate β-Klotho/FGFR1c signaling.** *A*, schematic representation of the Fab fragment of IgG-VH1+VH2. *B*, stimulation of Elk1-luciferase activity by Fab-VH1+VH2 in CHO reporter cells. *C*, binding of Fab-VH1+VH2 to human β-Klotho measured by bio-layer interferometry. *K_D_* values were estimated using a globally fitted 1:1 binding model. *D*, effect of Fab-VH1+VH2 on FGF21 signaling. CHO reporter cells stably expressing human β-Klotho and FGFR1c were treated with a combination of FGF21 and Fab-VH1+VH2 for 4 h, after which luciferase activity was measured. Data are representative of three independent experiments and expressed as mean ± S.E.

We further sought to understand the geometry required for receptor activation. Because IgG-VH1+VH2 is tetravalent for β-Klotho, the VH1 and VH2 pairs are present with two geometries, one being close with the two binding arms within each of the Fab fragment, and the other being distant with one binding arm from each of the two Fab fragments (Fig. 3*A*). To assess the relative contribution of these two geometries to receptor binding and activation, we generated a biparatopic Fab molecule, Fab-VH1+VH2 (Fig. 5*A*). As shown in Fig. 5*B*, Fab-VH1+VH2 activated β-Klotho/FGFR1c signaling, achieving ∼60% of the maximal response of IgG-VH1+VH2. Notably, the level of receptor activation by Fab-VH1+VH2 (36.7 ± 4.0% of FGF21 *R*_max_) was higher than that achieved by Fab-VH1+VH1 (18.6 ± 1.6%, *p* = 0.015) or Fab-VH2+VH2 (dose-response curve did not converge), further supporting the notion that both VH1 and VH2 arms contribute to the full agonistic activity of the biparatopic molecule. Compared with IgG-VH1+VH2, the Fab fragment exhibited a 10-fold reduction in binding affinity for β-Klotho as reflected by *K_D_* estimation (Fig. 5*C*), likely from a loss of avidity. The Fab molecule also inhibited FGF21 signaling (Fig. 5*D*), but this effect was partial compared to IgG-VH1+VH2, which may be because of the smaller size of the Fab molecule and thus, less steric hindrance.

## Discussion

Bispecific (or multispecific) antibodies have become critically important for therapeutic discovery in recent years. Bispecific molecules not only allow potential synergy by the simultaneous engagement of two separate targets, but also enable new functions that are not possible to achieve by simple combination of two monospecific antibodies, such as tissue-specific delivery and bispecific T cell engagers in immuno-oncology drug development (1, 2). Although antagonistic antibodies that disrupt protein/protein or ligand/receptor interactions predominate drug discovery, monospecific or bispecific antibodies capable of activating receptor signaling have also been identified (21). For receptors formed by two components, a bispecific molecule that engages both receptor components simultaneously may be required for optimal signaling. Therefore, bispecific antibodies may be particularly useful in activating multicomponent receptor complexes.

Many different approaches, both natural ligand-based as well as non–FGF-based analogs, have been explored for therapeutic development to target the FGF21 signaling pathway (12, 21). Two types of agonistic antibodies for the FGF21 receptor complex, β-Klotho/FGFR1c, have been reported. Compared with the natural ligand, all of these FGF21-mimetic agonistic antibodies achieved various levels of partial activation of the β-Klotho/FGFR1c complex. Because the epitope and geometry are particularly important for agonistic antibodies (17, 18) and the two types of FGF21-mimetic antibodies reported have large distances between the two binding arms compared with the native ligand FGF21, we explored here a new format with distinct geometry to activate the β-Klotho/FGFR1c receptor complex.

We termed this new format a biparatopic antibody because it is constructed by combining two different binders to two distinct epitopes on the β-Klotho co-receptor. Two noncompeting binders that bind to distinct epitopes on β-Klotho, VH1 and VH2, were identified and selected for further evaluation. VH1 and VH2 did not compete with FGF21 for binding to β-Klotho (Fig. 1). Neither binder activated the β-Klotho/FGFR1c receptor complex as VHO format, and only weak partial agonistic activity was observed for VH1 in the HCAb, Fab, and IgG formats and for VH2 in the IgG format (Fig. 2, *B* and *E*). These data demonstrate that simply cross-linking receptors is not sufficient for potent signaling, highlighting the importance of binding a specific epitope and adopting an optimal geometry in agonistic antibody design.

Our biparatopic format involves grafting one VH binder in place of VH and grafting the other VH binder in place of VL in an IgG format (Fig. 3*A*). The overall conformation of the final molecule resembles that of a typical antibody; this configuration presents two distinct geometries of the binding arms, one is a close geometry of the two different binding arms within each of the Fab fragment, and the second is the farther geometry of the different binders between the two Fab arms. In addition to presenting both a close and distant geometry between the two different binding arms, this format would also eliminate concerns of heterogeneity as a result of mispairing that has been observed with other bispecific formats.

This biparatopic IgG molecule (IgG-VH1+VH2) is active and demonstrates an activity level higher than the VHs by themselves in the VHO, HCAb, Fab, or IgG format (compare Fig. 3 to Fig. 2). The activity of the biparatopic IgG molecule depends on the presence of both VH1 and VH2, as the pairing of an unrelated VL to VH1 (IgG-VH1+sLC) in an identical format yielded no activity (Fig. 4). Given that VH1 in the HCAb (HCAb-VH1) and IgG format (IgG-VH1+VH1) partially activated β-Klotho/FGFR1c receptor signaling (Fig. 2), it is somewhat surprising that VH1 is inactive in the IgG format when paired with an unrelated light chain. This may be the result of steric hindrance or an altered geometry induced by the light chain that either prevented the efficient binding of VH1 to β-Klotho or yielded an unproductive receptor complex. Consistent with this, the affinity of IgG-VH1+sLC is reduced almost 100-fold compared with HCAb-VH1 and IgG-VH1+VH1. We conclude that the relative position of the two VH domains in the biparatopic antibody is important for activity. Additionally, VH1 grafted onto the VH position (IgG-VH1+VH2) is more active than when it was grafted onto the VL position (IgG-VH2+VH1) (Fig. 4), further demonstrating the importance of the relative geometry of the binding arms to agonistic activity. In this case, the particular geometry conferred by VH1 on the VH position and VH2 on the VL position might facilitate dimerization of β-Klotho and FGFR1c, thereby promoting signal activation. This is likely different from how the endogenous ligand FGF21 interacts with β-Klotho. Further information on the structure of β-Klotho and the epitope of VH1 and VH2 would be needed to better understand how and why the IgG-VH1+VH2 format resulted in a more favorable receptor conformation. It is likely that the most favorable geometry of an agonistic antibody is target dependent and each case would need to be determined experimentally.

Because our biparatopic IgG molecules are tetravalent for β-Klotho–binding domains, the agonistic activities observed could come from the close geometry (the two binding arms within each of the Fab fragment) or the distant geometry (one binding arm from each of the two Fab fragments). To distinguish between these two possibilities, we generated just the Fab fragment alone with VH1 fused to CH1 and VH2 fused to Cκ (Fab-VH1+VH2). Indeed, the Fab fragment alone is active (Fig. 5). Although its potency is slightly lower than the IgG format (IgG-VH1+VH2), Fab-VH1+VH2 demonstrates an activity higher than HCAb-VH1 or IgG-VH1+VH1. Although we cannot rule out the contribution by the distant geometry of the binding arms in IgG-VH1+VH2 to full agonistic activity, these results do suggest that the close geometry in the mini-biparatopic Fab version is sufficient for agonistic activity.

Although numerous bispecific formats have been described, a biparatopic antibody capable of agonizing a multicomponent receptor complex has not been reported. We have developed a novel biparatopic format that activates the endocrine FGF receptor complex containing β-Klotho and FGFR1c. This format presents two potential geometries, a close and a distant, between the two paratopes, and our data revealed that the biparatopic molecule provided novel activities not observed with each paratope alone. This format is versatile, easy to construct, and the single-domain binders could come from a variety of sources. This approach opens a new avenue for agonistic antibody generation against other therapeutically interesting cellular receptors.

## Experimental procedures

### Immunizations

To generate heavy chain–only antibodies (HCAbs) to human β-Klotho, cohorts of three Harbour 4HVH or 8V3 HCAb mice were immunized. These transgenic animals secrete HCAbs using a restricted set of human VH genes without cognate light chains (7). Animals were immunized with a stable AM-1/D CHO cell line expressing full-length human β-Klotho and human FGFR1c (19). 2 × 10^6^ cells were mixed with Alum (2% (w/v) Al(OH)_3_) and CpG-ODN (5′-T*C*C*A*T*G*A*C*G*T*T*C*C*T*G*A*C*G*T*T-3′ where * represents phosphorothionated bases) as adjuvant and delivered to animals alternating between a single dorsal subcutaneous site and an intraperitoneal injection. Mice were immunized twice per week for the first 4 weeks and then once weekly for 12 additional weeks. The final boost was delivered in PBS without adjuvants. Human β-Klotho–specific serum titers were monitored by live-cell fluorescence activated cell sorting (FACS) analysis on a FACSCalibur (BD Biosciences) flow cytometer using HEK 293T cells transiently transfected with a human β-Klotho expression vector. Animals with the highest antigen-specific serum titers were sacrificed and B cells were harvested from spleen, lymph nodes, and bone marrow for RNA extraction and yeast display library construction. Animal studies were approved by the Institutional Animal Care and Use Committee (IACUC).

### Yeast display

CD138^+^ cells from immune animals were enriched using positive selection with the EasySep^TM^ Mouse PE Positive Selection Kit according to the manufacturers' instructions (STEMCELL Technologies). The cells were lysed in RLT lysis buffer and total RNA was purified using an RNeasy Mini Kit (Qiagen). HCAb-specific cDNA was synthesized by reverse transcription–polymerase chain reaction (RT-PCR) using Qiagen OneStep RT-PCR Kit (Qiagen). A second round of PCR was carried out to add identical 5′ and 3′ sequences (5′-CTT GGG CAT CTA GAT ACC CAT ACG TAC CAG ATT ACG CTG GAT C-3′ and 5′-GAT CCT CTT CTG AGA TGA GTT TTT GTT CTG AAC CTC CAC CTC CGG TAC-3′, respectively) to aid in homologous recombination in yeast when co-transformed with an appropriate display vector carrying the same 5′ and 3′ sequences and a downstream agglutinin coding sequence (NCBI accession no. NP_012537.1, aa 330–650). The resulting VH-agglutinin fusion proteins were displayed on the surface of yeast (via agglutinin), and antigen-specific VH binders were enriched via FACS using biotinylated soluble antigen (the ECD of human β-Klotho (aa 1–992)) conjugated to streptavidin-APC (Thermo Fisher Scientific). Three rounds of FACS were carried out to enrich for human β-Klotho binders. Individual yeast colonies were then picked up for PCR amplification and sequencing analysis.

FACS-based competition experiments were used to screen for VH binders that did not inhibit the interaction of human β-Klotho with human FGFR1c or FGF ligands. Briefly, individual yeast clones displaying human β-Klotho–specific VH domains were tested for their ability to bind to human β-Klotho in the presence of excess competitor (10 μg/ml FGF19, FGF21, or FGFR1c D2-D3 (aa 142–365). Two of the noncompeting human β-Klotho–specific VH domains (VH1 and VH2) were selected for recombinant engineering and further analysis.

### Cloning, expression, and purification

Sequences corresponding to VH1 and VH2 in VHO (with a C′-terminal His_6_ tag), HCAb, Fab, or IgG formats were cloned into a pTT5 expression vector driven by a cytomegalovirus (CMV) promoter. All antibody formats were transiently transfected into HEK 293–6e suspension cells at 0.5 μg total DNA/ml culture during the exponential growth phase (1.1–1.6 × 10^6^ cells/ml). Cells were maintained in FreeStyle F17 Medium (Thermo Fisher Scientific) supplemented with 0.1% (w/v) Poloxamer 188 (Sigma-Aldrich), 6 mm GlutaMax (Thermo Fisher Scientific), 25 μg/ml G418 (Thermo Fisher Scientific) without feed. Conditioned medium was harvested 6 days post transfection by centrifuging cells at 3485 × *g* for 30 min and filter sterilizing the supernatant with 0.2 μm polyethersulfone filter. If not used immediately for affinity purification, conditioned medium was stored at −80 °C.

His_6_-tagged VHO and Fab antibodies were purified from conditioned medium using Ni Sepharose excel (GE Healthcare) and eluted with 20 mm Tris-HCl, pH 7.0, 150 mm NaCl, 0.5 m imidazole, whereas Fc-containing HCAbs and IgGs were purified from conditioned medium using MabSelect SuRe (GE Healthcare) and eluted with 0.5% (v/v) acetic acid, pH 3.5, 150 mm NaCl and neutralized with 10% (v/v) of 1 m Tris-HCl, pH 8.0. Purification schema was performed according to manufacturer's protocols. Concentrated eluate fractions were pooled and buffer exchanged into PBS, pH 7.4, polished by size-exclusion FPLC (HiLoad 16/600 Superdex 75/200 column, GE Healthcare), and peak fractions chosen, prior to use in downstream assays.

Full-length FGF21 protein was expressed and purified from an *Escherichia coli* expression system as described previously (19). The ECD of human β-Klotho (aa 1–992) fused with a His_6_ tag at the C terminus was cloned into the pTT14 expression vector and stably expressed in HEK 293F cells. β-Klotho protein was purified by Ni-IMAC on a HisTrap FF Crude column (GE Healthcare), and further purified on a HiLoad 26/60 Superdex 200 column (GE Healthcare) as described previously (22).

### L6 cell culture, transfections, and analysis of pERK activation

L6 rat myoblast cells were maintained in Dulbecco's modified Eagle's medium (Mediatech) supplemented with 10% (v/v) fetal bovine serum (Invitrogen) and 1× penicillin/streptomycin (Invitrogen) in a 37 °C incubator with 5% CO_2_. Cells were plated in 96-well plates, and 24 h post plating, cells were transfected with expression vectors encoding human β-Klotho and individual human FGFR isoforms using the Lipofectamine 2000 transfection reagent (Invitrogen) according to the manufacturer's protocol. FGFR and β-Klotho were previously cloned into the pcDNA3.1(+)-hygromycin (Invitrogen) and pTT14 vectors, respectively. Cells were serum-starved in Dulbecco's modified Eagle's medium supplemented with 0.2% (w/v) BSA overnight the day after transfection and treated with vehicle (PBS) or test molecules for 10 min. Following treatment, the medium was aspirated and cells were snap frozen in liquid nitrogen. Cell lysates were prepared and total ERK and pERK levels were measured with the Phospho/Total ERK1/2 Whole Cell Lysate Kit (Meso Scale Discovery) according to the manufacturer's instructions. All experiments were run in duplicate.

### Bio-layer interferometry

Binding of VHO, HCAb, Fab, and IgG molecules to β-Klotho or FGFR1c was measured by bio-layer interferometry on an Octet RED instrument (Pall ForteBio). Briefly, β-Klotho ECD His_6_ was biotinylated with EZ-Link Sulfo-NHS-LC-Biotin (Pierce). Ten micrograms per ml of biotinylated β-Klotho protein in kinetics buffer were loaded onto streptavidin biosensors (ForteBio). Recombinant human FGFR1c ECD Fc chimeric protein (R&D Systems) was loaded onto anti-human IgG Fc biosensors (ForteBio). After an initial baseline step for 2 min in kinetics buffer, VHO, HCAb, Fab, or IgG molecules were allowed to associate with the immobilized β-Klotho at various concentrations for 5 min, followed by a dissociation step for 10 min in kinetics buffer. Blank binding cycles containing no antibody were included to correct for baseline drift. Data were fitted globally to a 1:1 binding model using ForteBio Octet Data Analysis Software version 7.1 and *K_D_* values were calculated. For cross-competition of VHOs and FGF21, 10 μg/ml of biotinylated β-Klotho protein in kinetics buffer was loaded onto streptavidin biosensors. VHOs or FGF21 was allowed to associate with the immobilized β-Klotho for 5 min, followed by association of the competing analyte for 5 min. Data were obtained from at least three independent experiments.

### Luciferase reporter assay

Luciferase reporter assays were performed in CHO cells stably transfected with human FGFR1c, β-Klotho, and reporter constructs containing 5× Upstream Activation Sequence upstream of luciferase and Gal4 DNA-binding domain fused to Elk1 as described previously (19). CHO reporter cells were maintained in Dulbecco's modified Eagle's medium supplemented with 10% (v/v) dialyzed fetal bovine serum (GE Healthcare), 6 μg/ml puromycin, and 400 μg/ml hygromycin in a 37 °C incubator with 5% CO_2_. Cells were plated at 2.5 × 10^4^ cells/well on 96-well plates in growth medium and incubated overnight at 37 °C with 5% CO_2_. The next day, the medium was replaced with starvation medium (Ham's F-12K Nutrient Mixture plus 1% (w/v) BSA) and the cells were incubated overnight at 37 °C with 5% CO_2_. Following starvation, treatment molecules were added to the cells in starvation medium in duplicate. The plates were incubated for 4 h at 37 °C with 5% CO_2_, after which the cells were lysed in Steady-Glo luciferase reagent (Promega). After a 45-min incubation at room temperature, luciferase activity was measured in relative luminescence units on the EnVision Multilabel Plate Reader (Perkin Elmer) according to the manufacturer's instructions.

### Statistical analysis

Dose-response curves were fitted from a three-parameter logistic regression model with the least squares fit using GraphPad Prism version 7.02 (GraphPad Software). Data are presented as mean ± S.E. Values were analyzed by two-tailed unpaired Student's *t* test using GraphPad Prism version 7.02. *p* values < 0.05 were accepted as statistically significant.

## Author contributions

S. Y. S., Y.-W. L., and V. W. data curation; S. Y. S. and Y.-W. L. formal analysis; S. Y. S. visualization; S. Y. S., Y.-W. L., and Y. L. writing-original draft; S. Y. S., Y.-W. L., Z. L., J. S., C. M. M., W. G. R., M. L. M., J. Z., W. Y., and Y. L. writing-review and editing; Z. L., J. S., C. M. M., V. W., Z. H., W. G. R., M. L. M., and W. Y. resources; C. M. M., W. G. R., M. L. M., J. Z., W. Y., and Y. L. supervision; M. L. M., W. Y., and Y. L. conceptualization.

## Footnotes

3 The abbreviations used are:

VH: variable domain heavy chain
VL: variable domain light chain
VHH: variable domain of camelid heavy chain-only
HCAb: heavy chain-only antibody
CL: constant domain light chain
CH: constant domain heavy chain
FGFR: fibroblast growth factor receptor
ECD: extracellular domain
Cκ: constant domain kappa light chain.
